# Retraction: Pediatric Acute Dacryocystitis and Orbital Cellulitis With Concurrent COVID-19 Infection: A Case Report

**DOI:** 10.7759/cureus.r147

**Published:** 2024-08-14

**Authors:** Saleh Ghulaysi, Fatima Alhumaid, Manar Almania, Nouf AlQurashi, Ahmed Abdelaziz

**Affiliations:** 1 General Practice, Jazan University, Jazan, SAU; 2 General Practice, Imam Abdulrahman Bin Faisal University, Dammam, SAU; 3 General Practice, Majmaah University, Al-Majma'ah, SAU; 4 General Practice, King Saud bin Abdulaziz University for Health Sciences, Riyadh, SAU; 5 Ophthalmology, Dallah Hospital, Riyadh, SAU

This article has been retracted by the Editor-in-Chief due to plagiarism. The images presented in Figure 1 have been plagiarized from an online lecture that was uploaded to the internet, but removed by the original author after discovery of the plagiarism. This author, who has requested to remain anonymous, provided the journal with the original images. In addition, the authors of this article were unable to verify the authenticity of the patient data upon request. As a result, this article has been retracted.

